# Enhancing the quality of panel-based tumor mutation burden assessment: a comprehensive study of real-world and in-silico outcomes

**DOI:** 10.1038/s41698-024-00504-1

**Published:** 2024-01-23

**Authors:** Yuanfeng Zhang, Duo Wang, Zihong Zhao, Rongxue Peng, Yanxi Han, Jinming Li, Rui Zhang

**Affiliations:** 1 National Center for Clinical Laboratories, Institute of Geriatric Medicine, Chinese Academy of Medical Sciences, Beijing Hospital/National Center of Gerontology, Beijing, PR China; 2 National Center for Clinical Laboratories, Chinese Academy of Medical Sciences & Peking Union Medical College, Beijing, PR China; 3 Beijing Engineering Research Center of Laboratory Medicine, Beijing Hospital, Beijing, PR China; 4 Peking University Fifth School of Clinical Medicine, Beijing, PR China

**Keywords:** Cancer genomics, Next-generation sequencing, Cancer genomics, Tumour biomarkers

## Abstract

Targeted panel-based tumor mutation burden (TMB) assays are widely employed to guide immunotherapy for patients with solid tumors. However, the accuracy and consistency of this method can be compromised due to the variability in technical details across different laboratories, particularly in terms of panel size, somatic mutation detection and TMB calculation rules. Currently, systematic evaluations of the impact of these technical factors on existing assays and best practice recommendations remain lacking. We assessed the performance of 50 participating panel-based TMB assays involving 38 unique methods using cell line samples. In silico experiments utilizing TCGA MC3 datasets were performed to further dissect the impact of technical factors. Here we show that the panel sizes beyond 1.04 Mb and 389 genes are necessary for the basic discrete accuracy, as determined by over 40,000 synthetic panels. The somatic mutation detection should maintain a reciprocal gap of recall and precision less than 0.179 for reliable psTMB calculation results. The inclusion of synonymous, nonsense and hotspot mutations could enhance the accuracy of panel-based TMB assay. A 5% variant allele frequency cut-off is suitable for TMB assays using tumor samples with at least 20% tumor purity. In conclusion, this multicenter study elucidates the major technical factors as sources of variability in panel-based TMB assays and proposed comprehensive recommendations for the enhancement of accuracy and consistency. These findings will assist clinical laboratories in optimizing the methodological details through bioinformatic experiments to enhance the reliability of panel-based methods.

## Introduction

Immune checkpoint inhibitors (ICIs) targeting PD-1 and PD-L1 have considerably transformed the treatment landscape of solid tumors^1,2^. Tumor mutation burden (TMB), a biomarker that quantifies the number of somatic mutations in a cancer genome, is progressively utilized in clinical next-generation sequencing (NGS) centers for predicting patients’ response to immunotherapy and their prognosis^3,4^. Specially, the large targeted panel-based TMB assessment method is highly favored not only for its cost and time advantages compared to the gold standard—whole exome sequencing (WES), but also for its strong consistency with WES results^5,6^. An international survey conducted by the International Quality Network for Pathology (IQN Path) revealed that the targeted panel-based assay has in fact become the predominant method for TMB measurement in research and/or clinical applications^7^.

**Fig. 1:**
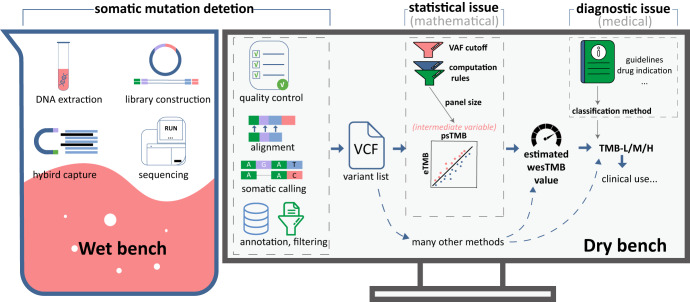
Workflow of panel-based TMB assay. The whole process can be divided into three parts: somatic mutation detection based on targeted sequencing, estimation of wesTMB values (statistical issue) and classification of TMB results (diagnostic issue).

However, despite the promising potential of panel-based TMB analysis, the process of this method is technologically complex and composed of three sequential yet distinct parts (Fig. 1)^8–10^. The first stage involves somatic mutation detection, which includes multiple steps from DNA extraction to variant calling and filtering. The two subsequent stages entail the statistical estimation of TMB level and diagnostic prediction of response to ICIs. In the statistical estimation stage, the count of selected somatic mutations is used to calculate the psTMB value, utilizing multiple computation rules including mutation classifications, filtering rules and variant allele frequency (VAF) cut-off. The psTMB value can further be converted into a wesTMB value through a simple regression model, thereby representing the status of the whole genome. In the diagnostic stage, samples are classified as TMB-high/low/medium (TMB-H/L/M) using the wesTMB values. Technical details from the process may contribute to the variability of TMB assessment, including the methodologies of somatic mutation detection, psTMB calculation rules, panel size and TMB-H cut-off. Given the absence of corresponding consensus and guidelines, the accuracy and consistency of laboratory-developed panel-based assays cannot be guaranteed. In essence, there is an urgent need to clarify the impact of major factors on TMB assessment and propose recommendations to solidify the future of the panel-based TMB assessment in clinical settings.

A few standardization and harmonization studies have been conducted to evaluate the performance of several commercial panel assays and initially measure the influence of panel size and calculation rules on panel-based TMB assessment^10–29^. For instance, the Friends of Cancer Research (FOCR) proposed fundamental recommendations for the standardization^20^. Subsequently, in its second phase, the impact of certain factors on the accuracy of panel-based TMB analysis was assessed, such as panel size, the inclusion of synonymous mutations and population minor allele frequency (pMAF) threshold^26^. Another collaborative study by FOCR and Quality in Pathology (QuIP) evaluated the impact of classification method, performance metrics and sample DNA quality on the accuracy and consistency of several panel-based assays^22^. Generally, there is a broad consensus that the panel assay should cover at least about 1 Mb of the exonic region. However, the impact of somatic mutation detection process and VAF cut-off, also a part of psTMB calculation rules, have yet been explored. Furthermore, there is currently no consensus regarding other methodological details. Evidence for this lack can be found in the IQN Path pilot program^7,30^. This pilot program highlighted the discrepancies of methodologies and performance among different methods, especially the inclusion of synonymous mutations, among various commercial and laboratory-developed panel assays, suggesting a need for further investigation.

**Fig. 2:**
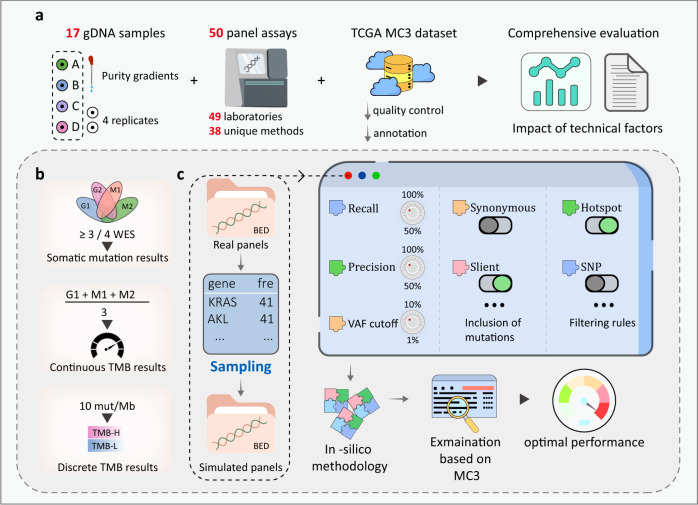
Comprehensive study design for evaluating the key technical factors. **a** 17 tumor samples derived from four edited clones and a shared paired sample were prepared as the sample panel for real-world assessment. There were 50 panel-based TMB assays from 54 laboratories participating this study. The MC3 dataset was cleansed for the in silico experiment. **b** In real-world assessment, the truth set of somatic mutation, continuous and discrete wesTMB results of the gDNA samples were established using four WES assays and compared with the results of 50 panels to assess their performance of somatic mutation detection and TMB estimation. **c** Simulated experiment was used to explore the theoretically best performance of the real panels and synthetic panels with selected parameters.

Hence, to assess the impact of aforementioned technical factors on TMB detection accuracy, we conducted a large-scale multicenter study based on a series of CRISPR-edited 293T subclones. These subclones and original 293T cell line were used to simulate tumor and paired normal samples (Fig. 2a). The mutation truth set was established using multiple ultra-deep WES assays (Fig. 2b). The methodologies and detection results of 50 participating laboratories on these genomic DNA (gDNA) samples were evaluated (Fig. 2b). Due to the limitations in sample size and confounding factors, we further measured the importance of technical factors through in silico experiment (Fig. 2c), based on the Multi-Center Mutation Calling in Multiple Cancers (MC3) dataset (Fig. 2a). Furthermore, we focused on individual technical factors to understand their effects. Specifically, for panel size, we assembled over 40,000 synthetic panels mirroring the design patterns of real panels to depict the efficiency curves (Fig. 2c). For VAF cut-off, the performance of different cut-off values (1–10%) under varying tumor purities were explored. Based on our findings, we detailed the impacts of various technical factors on the TMB detection accuracy, and proposed recommendations for quality enhancement and comparability assurance.

## Results

### Methodologies and TMB results of panel assays

Out of the 38 panel methods evaluated from 50 submitted results, 28.9% (11/38) have been widely used in clinical studies. Six of these methods were employed more than once, with p10 OncoScreen Plus (Burning Rock Dx) and p7 Onco1021plus (GenePlus Technology) being the most frequently utilized methods. All the TMB assays were laboratory-developed tests expressly for scientific research purpose and not for clinical decision. The details of enrolled panel methods can be found in Supplementary Table 2.

The wet-bench experiments of the submitted panel assays showed significant similarities (Table 1). The majority of panels (33/38, 86.8%) covered exon regions of more than 1 Mb and 400 to over 1500 genes. The prevalent VAF cut-offs for somatic mutation calling and TMB calculation were both 5%. Nonsense, missense, and small insertions and deletions (indel) (both frameshift and in-frame) were the basic variants that involved in the psTMB calculation by all panel assays. Synonymous mutations were utilized only by a subset of panels (13/38, 34.2%).

**Table 1:** Description of 38 participating NGS panel methods.

Methodology	Method count	Methodology	Method count
Exon size (Mb)		Involved mutations	
0–1.0	5	Nonsense	38
1.0-2.0	26	Missense	38
≥2.0	7	Indel	38
Gene count		Nonstop	35
400–500	1	Splicing site	29
500–600	13	Synonymous	13
600–700	14	Other	4
700–1000	6	Applied filtering rules	
1000–1600	4	SNP	29
Somatic VAF cut-off (%)		Driver	19
1	12	Hotspot	11
2	7	Known	7
3	1	Other	7
5	18	TMB-H cut-off (mut/Mb)	
TMB VAF cut-off (%)		5–10	15
1	1	10	11
2	4	10–11	8
5	26	11–21	4
10	1	Classification method	
Other	6	Binary (TMB-L, H)	36
Classification stage		Ternary (TMB-L, M, H)	2
psTMB	23		
eTMB (wesTMB)	13		
Unstated	2		

The slope of the psTMB-wesTMB linear model of panel-based method, which can reflect the differences among panels, was influenced by factors such as panel design and calculation rules. Most panels (27/38, 71.1%) having slopes less than 1.0. Furthermore, over half (16/27, 59.3%) of these panels exhibited slope values less than 0.9, suggesting a propensity for most panel methods to overestimate TMB values at the psTMB level.

The majority of panels (36/38, 94.7%) employed binary classification method and half (19/38, 50.0%) used a binary cut-off close to 10 mut/Mb. The TMB results were classified at psTMB stage and estimated wesTMB (eTMB) stage by 33 panels (23/38, 60.5%) and 13 panels (13/38, 34.2%), respectively. This indicated divergent interpretations of panel-based TMB assessment.

**Fig. 3:**
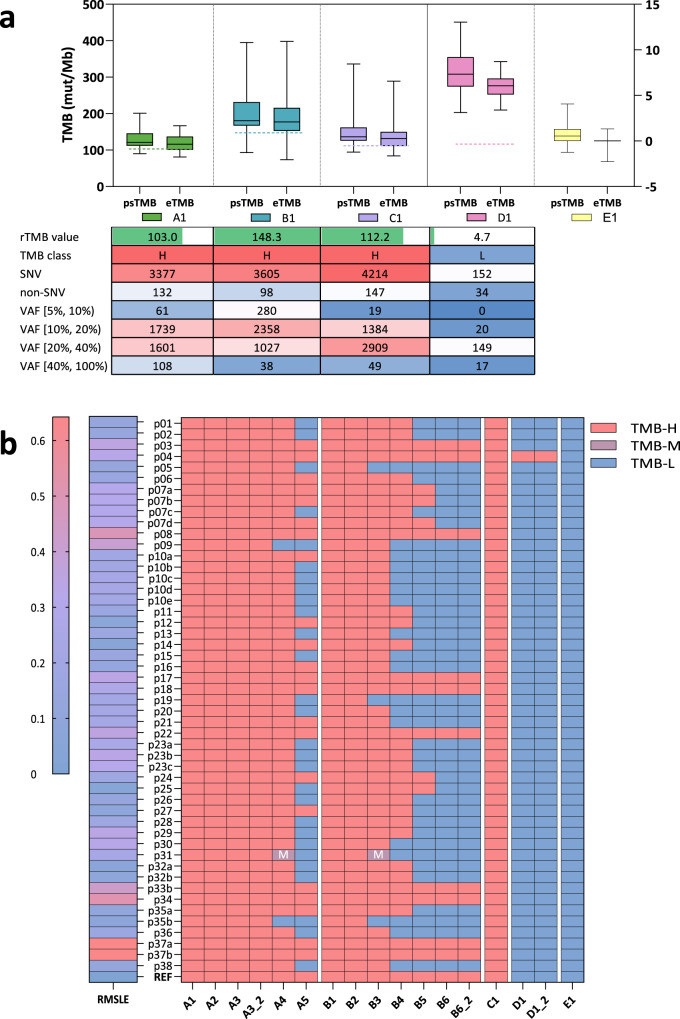
TMB values and classification results submitted by 50 participating panels. **a** The distribution of psTMB and estimated wesTMB values on five undiluted samples, including A1, B1, C1, D1 and E1 (negative control). Dotted lines show the reference TMB values. The chart below shows the characteristic of truth set. **b** Continuous and discrete TMB results of panels. The continuous TMB results were calculated using the samples with at least 40% simulated tumor purity. The bottom REF row shows the classification results confirmed by WES assays, with a binary 10 mut/Mb threshold.

Most panels correctly classified original samples (A1, B1, C1, D1, E1) and diluted samples with 40% tumor purity (A2, A3, and B2), but they did not perform well on samples with lower tumor purity (A5, B4, B5, and B6). Moreover, participating panels tended to markedly overestimate the TMB levels, leading to a dispersed distribution of psTMB compared to eTMB across panels (Fig. 3a). The root mean squared logarithmic error (RMSLE) ranged from 0.05 to 0.64, with a median of 0.21, indicating considerable variation in continuous accuracy among panels (Fig. 3b). The generally inflated TMB values and inconsistent RMSLE values suggested the need for further investigation into the accuracy of panels. Performance details and methodologies of all panels can be found in Supplementary Tables 1 and 2.

**Fig. 4:**
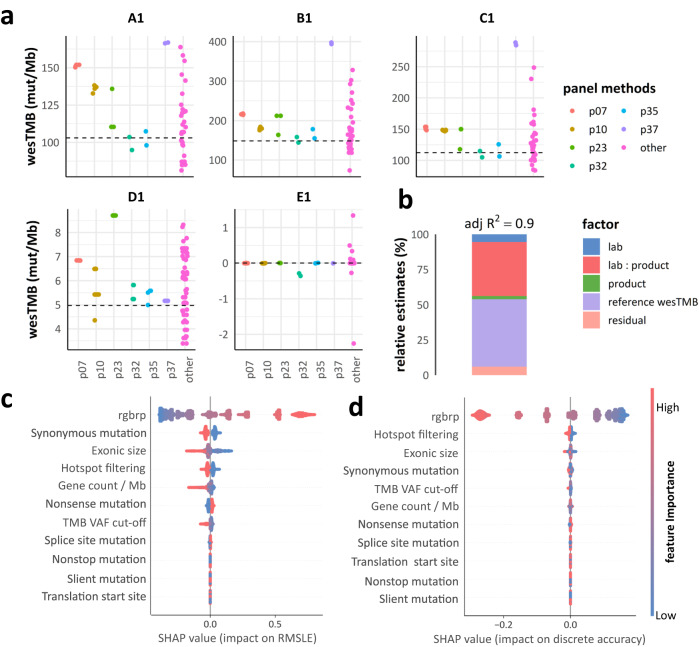
Interaction between lab and panel product, and SHAP values of key factors. **a** The eTMB results of different panel methods on the five undiluted gDNA samples. Methods only used by one lab are gathered as “other”. The reference wesTMB values of these samples are shown as horizontal dotted lines. **b** The stacked barplot right side from AVOVA shows the relative contributions of listed factors to the eTMB results. **c** SHAP values of technical factors on the continuous accuracy of panel-based TMB methods. **d** SHAP values of technical factors on the discrete accuracy of panel-based TMB methods. Technical factors are ranked by the relative importance from top (most important) to bottom.

There were six unique methods used by more than one laboratory, including p07, p10, p23, p32, p35, and p37. The intra-method concordance was high among the panels sharing p07 and p37 (Fig. 4a). The analysis of variance (ANOVA) results showed that the concordance varied among the methods, and the interaction between laboratory and method was critical for the TMB accuracy (Fig. 4b). There was no significant performance difference observed between the results from the 12 panel methods that have been used in publish clinical studies and other panel methods (*t*-value = 0.254, *p* value = 0.801, effect size = 0.013).

### Relative importance of features

The SHapley Additive exPlanations (SHAP) values indicated that among the measured features, the mutation detection accuracy (reciprocal gap between recall and precision) (rgbrp), panel size (composed of exonic size and gene count), the inclusion of synonymous mutations, and hotspot mutation filtering were approximately the most important features (technical factors) for the performance of panel-based TMB estimation (Fig. 4c, d). However, the detailed influences of these factors need further exploration. The SHAP values on the *R*^2^ can be found in Supplementary Fig. 1.

### Assessment of panel size

Given that psTMB calculation typically encompasses only the exonic regions, the term “panel size” within this article is exclusively used to denote the sizes of the exonic regions covered by the panels. The panel sizes of participating panels were concentrated (Table 1), and the correlation between panel size and TMB accuracy was not observed in the real-world results (Fig. 3b).

**Fig. 5:**
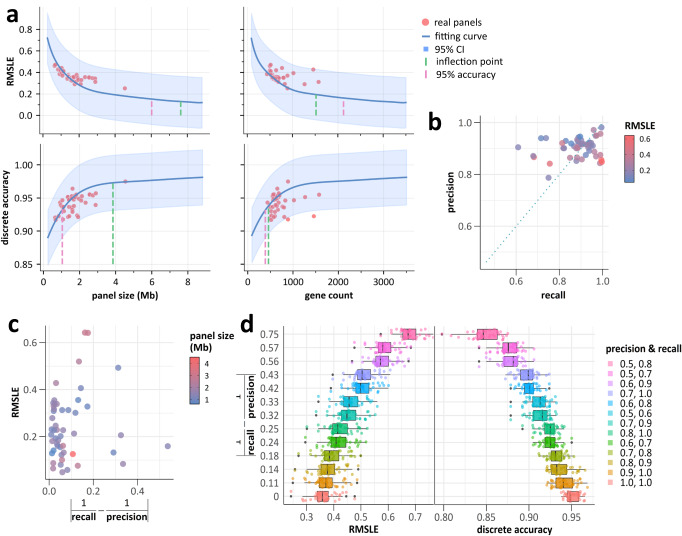
Detailed influence of panel size and mutation detection accuracy. **a** Impact of panel size and gene count on RMSLE and discrete accuracy of panel-based TMB assessment. The fitting curves represent the synthetic panels. The red dots represent the real panels. Confidence intervals of 95% are indicated by the light blue shadow. Critical panel size and gene count values (inflection points) and 95% theoretically best accuracy are shown as vertical lines. **b** Accuracy of panels on cell line-derived gDNA samples. **c** Distribution of RMSLE and the reciprocal gap between recall and precision on gDNA samples. **d** Impact of precision and recall value pairs on RMSLE and discrete accuracy of panel-based TMB assessment.

The results from over 40,000 synthetic panels showed a significant impact of panel size on both the continuous and discrete accuracy of panel-based TMB estimation (Fig. 5a). Specifically, if the panel size was less than 6.02 Mb and/or 2126 genes, it failed <95% of the theoretical optimal continuous accuracy. The thresholds for keeping 95% discrete accuracy were 1.04 Mb and 389 genes. In terms of cost-effectiveness, a decrease in the additional benefit of continuous accuracy per Mb and per gene was observed above 7.63 Mb and 1518 genes. The global inflection points for discrete accuracy corresponded to 3.85 Mb and 457 genes.

Our findings suggested that RMSLE was more sensitive than discrete accuracy, and the acceptable continuous accuracy required larger panel size than discrete accuracy. When focusing solely on discrete accuracy, our results were consistent with previous studies in that only about 1 Mb of exonic regions is necessary for 95% optimal accuracy. However, as, previous studies indicated that the current 10 mut/Mb threshold might not suit all patient groups^31,32^, laboratories should also ensure the continuous accuracy for the wide clinical application prospects of panel-based TMB methods.

In this analysis, certain real panel-based methods exhibited inferior performance when compared to the synthetic panels. This could be attributed to the many complex issues to be considered and balanced during the development of targeted large panel products, such as GC content and specificity of probes.

### Assessment of mutation detection accuracy

The majority of panel results demonstrated recall (43/50, 86.0%), precision (49/50, 98.0%), and F1 score (45/50, 90.0%) values over 0.8 in somatic mutation detection (Fig. 5b). The balance of mutation detection performance was measured by the rgbrp, with most (45/50, 90.0%) panel results exhibiting it below 0.2, indicating balanced performance (Fig. 5c). However, these panel results exhibited diverse continuous TMB accuracy with varying RMSLE values, suggesting the influence of other major technical factors beyond (Supplementary Table 1).

Germline sequencing of tumor-normal assays is critical to filter the mutations unrelated to the neoantigen potential. There were in total of only 5 false positive records from 6% (3/50) submitted results on the negative control sample E1, suggesting generally reliable germline sequencing.

The in silico results (Fig. 5d) showed that for every panel, the methodology with the modest yet balanced precision and recall values outperformed the methodology with high yet imbalanced combinations. For example, the RMSLE of panels with precision of 0.7 and recall of 0.8 was close to the ideally best RMSLE whilst being smaller than the RMSLE from panels with precision of 0.8 and recall of 1.0. The results of discrete accuracy were similar. These results indicated that an imbalanced somatic detection, such as striving for “zero false positive”, could create a disparity between false positives and false negative counts, thus affecting the TMB accuracy.

### Assessment of computation rules

Beyond the fundamental missense and small indels, synonymous mutations were least commonly included (13/38, 34.2%), while nonsense (38/38, 100%), nonstop (35/38, 92.7%) and splicing site mutations (29/38, 76.3%) were included in most panels. For filtering rules, single nucleotide polymorphisms (SNPs) were commonly filtered (29/38, 76.3%), while known mutations were generally not (7/38, 18.4%). While the assays incorporating synonymous mutations performed better than those not (*t*-value = 2.199, *p* value = 0.033, Cohen’s *d* = 0.611), the other computation rules did not show significant impact on the performance (Supplementary Table 3).

**Fig. 6:**
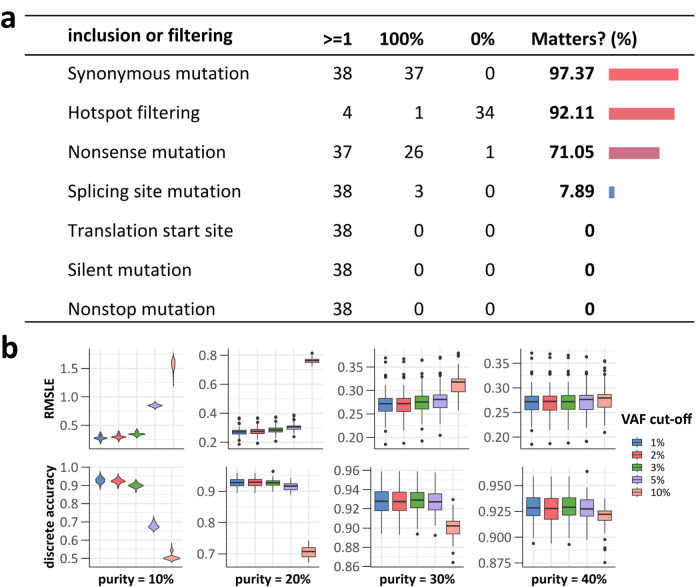
Detailed influence of computation rules and VAF cut-off. **a** Importance of variant classifications and filtering rules. The “≥1” column describes the number of panels which had at least one parameter combination with top performance including this rule. The “100%” column describes the number of panels for which all the parameter combinations with top performance included this rule. The “0%” column describes the number of panels for which all the parameter combinations with top performance all excluded this rule. The “Matters? (%)” column describes the percentage of panels that significantly affected by this rule. **b** Panel performance with five VAF cut-offs under four different tumor purity conditions.

The separate simulation results were consistent with the SHAP values (Fig. 6a). The order of importance was as follows: synonymous mutations > hotspot filtering > nonsense mutations > splicing site mutations. Most panels performed better when incorporating synonymous (37/38, 97.37%) and nonsense mutations (26/38, 68.42%) without filtering hotspot mutations (34/38, 89.47%). Splicing site mutations mattered for only a small subset (3/38, 7.89%) and can be optional. There was no significance of translation start site, silent and nonstop mutations observed.

### Assessment of VAF cut-off

A low VAF cut-off helps improve TMB reliability, especially on low-purity samples, but also present detection challenges, potentially affecting TMB assessment. While most (26/38, 68.0%) panel methods used a 5% VAF cut-off for psTMB calculation, some (11/38, 28.9%) used lower cut-offs, and their continuous TMB accuracy was polarized. Two panels (p25, p27) using a 2% cut-off exhibited continuous TMB accuracy, but six (p03, p08, p33, p34, p37a and p37b) of the seven panels with the worst performance used VAF cut-offs below 5%. Therefore, it was plausible that the aggressive VAF cut-off settings of those six methods indirectly influenced their TMB assessment.

In silico experiment revealed that the VAF cut-off did influence the TMB assessment on samples with low purity. However, the performance of 10% and 5% was comparable to that of lower cut-offs on samples with tumor purity ≥40% and 20%, respectively (Fig. 6b), and were insufficient only for sample with lower tumor purity. For tissue-based panel assays, which usually require a tumor purity of at least 20%, a 5% VAF cut-off is sufficient in capturing substantial somatic mutation data for TMB assessment. Lower cut-offs, while not enhancing TMB accuracy significantly, may adversely impact the somatic mutation results.

## Discussion

Panel-based TMB assessment has long been lacking anchors and standardization, with the methodologies being quite diverse^7–10,33^. For example, the inclusion of synonymous mutations has been a controversial topic^7,10,12–14,19,23,26^. In addition, it has been suggested that the ideal TMB-H cut-off may vary among certain patient populations^31,32,34,35^. Except for the indication of pembrolizumab for adults and children, there is no accepted classification and cut-off among panel assays, not even a consensus on the stage at which to classify. The aim of this study was to elucidate the factors affecting the TMB accuracy and address issues of quality enhancement and consistency by analyzing their essence. Specifically, serving as the most comprehensive standardization study of panel-based TMB analysis to date, this study evaluated the performance of 50 panel results from 38 unique panel methods. Furthermore, it objectively determined the impact of other major technical factors on TMB assessment via unbiased in silico experiment.

Our survey and the IQN Path’s pilot program agree that the panel assay has become the mainstream TMB approach, with many laboratories developing own methods^7,30^, and certain differences exist in the current methodologies. However, in this study, the relatively high intra-method consistency was observed for only 2 of 6 such methods. The limited data cannot directly determine the relative superiority of these panel products but only suggest their stability, and it is worth noting that directly discerning the impact of technical factors from real-world data was challenging. First, the technical factors of panel methods were largely similar. Second, the sample size may obscure the performance differences due to various factors. To measure the impact of factors objectively, we employed MC3 dataset with sufficient tumor samples and mutations, allowing for comprehensive and reliable exploration of the impacts of technical factors.

Despite the unavailability of FDA-proved F1CDx and MSK-IMPACT for us at that time, this study still incorporated several widely recognized panel products which have been extensively used in clinical trials, with or without validation reports (Supplementary Table 2), showing their predictive value for immunotherapy and prognosis of solid tumors^36–38^, and ensuring the validity and generalizability of our results. In silico experiment of all possible technical parameter combinations was conducted to measure the importance of them. Also, thousands of synthetic panels were assembled based on the real-world panel designs, instead of random sampling, allowing us to provide practical and applicable advice. Furthermore, our study focused on the mutation count and highlighted the intimate relationship between the balance of somatic detection strategy and psTMB calculation. In addition, RMSLE was used as the metric for TMB continuous accuracy since it is more applicable for skewed TMB distributions among patient populations than root mean square error (RMSE) and others. These improvements allow us to outline principles for factors influencing the panel-based TMB assessment and methodology development.

The results of in silico experiment indicated that a panel size above 1.04 Mb (389 genes) is necessary for basic discrete accuracy and a panel size of 6.02 Mb (2126 genes) is worth consideration for reliable continuous accuracy regardless of the TMB-H cut-off. It should be noted that he recommended values were based on the assumption that all exons were included. In practice, a more robust approach might be to moderately increase the panel size beyond them, and compare the performance of existing targeted region and synthetic panels covering all exons through further bioinformatic experiments.

Since the mainstream panel-based TMB method depends on the mutation count rather than any specific genomic site, it is essential to balance of false positive and false negative errors, which has been discussed in our previous study^39^. In this study, it was emphasized that the balance between precision and recall was more critical than excessively pursuing a higher value for either metric. For example, given the same recall of 0.7, a precision of 0.9 resulted in worse accuracy of TMB assessment than a precision of 0.6.

This large-scale study assured the importance of synonymous, nonsense mutations and hotspot mutations on the TMB accuracy. Specifically, the impact of synonymous mutations was consistent in external quality assessment (EQA) analysis and in silico experiment. For most panels, it’s commonly suggested to incorporate synonymous and nonsense mutations, without filtering hotspot mutations. We also found that the inclusion of other mutations will not significantly affect the TMB accuracy.

The VAF cut-off affected the TMB assessment in two distinct ways. First, it directly affected the TMB assessment only on low tumor purity samples. When the tumor purity dropped to 30–40%, the performance with a 10% VAF started to decline. However, the 5% VAF cut-off could still accommodate the samples with 20% tumor purity, meeting the basic pre-analysis quality control requirement for the most panel-based assays. Second, lower VAF cut-offs significantly increase the difficulty of somatic discovery^39,40^, making the 5% cut-off a reasonable and feasible choice.

According to the definition of TMB, only the number of mutations across the entire genome (wesTMB) is directly associated with the response to ICIs and prognosis. In contrast, the psTMB representing only a small portion of the cancer genome may not be able to replace the wesTMB. However, in this study, 23 panels (23/38, 60.5%) classified the TMB results at psTMB stage. Furthermore, clinical outcomes of local samples (8/38, 21.1%), wesTMB (21/38, 55.3%) and FDA-approved panel (4/38, 10.5%) were utilized as the reference standards for establishing the classification cut-off (Supplementary Table 2). There were 11 panel methods used other standards to determine the cut-off, such as the 75% quartile wesTMB value of the local clinical samples. As previously mentioned, while the TMB-H cut-offs may vary among various patient groups, it does not suggest that laboratories should develop own gold reference standards without the wesTMB. Instead, it requires a greater emphasis on the continuous accuracy which is independent of the TMB-H cut-off, in order to ensure robust clinical reporting. We also recommend laboratories uniformly adopt their TMB-H cut-offs and classify the TMB results at the estimated wesTMB stage. This practice would ensure essential comparability and consistency among panel-based TMB results.

A limitation of our study is the absence of commonly used clinical formalin-fixed and paraffin-embedded (FFPE) samples. Certain characteristics of FFPE samples such as tumor heterogeneity and fixation artifacts can affect the accuracy of mutation and TMB detection^13,16^, but our study concentrated on the primary technical factors and did not address this issue. We intend to incorporate clinical cases and explore sample features in subsequent study for a more thorough and realistic evaluation of panel-based TMB assays. Additionally, it should be noted that this study focused on the mainstream panel-based TMB methods for pan-cancer scenarios or non-small cell lung cancer. And the conclusions may not apply to certain tumor types with unsatisfactory TMB effectiveness^31,32^, such as renal cell carcinoma and mesothelioma. For these tumor types, further separate study is need to explore the alternative strategies, such as the neoantigen burden based on structural variants and chromosome arrangements^41,42^.

There are some recommendations that can help laboratories improve the quality of panel-based TMB assays (Table 2). Although current panel sizes are sufficient for TMB classification, they fall short for reliable TMB values, questioning their application on different patient groups with potential varying TMB-H cut-offs. It is also suggested for laboratories to include the synonymous and nonstop mutations, without filtering the hotspot mutations. For tissue samples, the 5% VAF cut-off is ideal for psTMB calculation. TMB results should be classified using estimated wesTMB for comparability.

**Table 2:** Evidence-based recommendations for quality enhancement of panel-based TMB assessment.

Methodology checklist	Recommendations
1. Panel size	
	• Discrete accuracy: a panel size above 1.04 Mb (389 genes) is necessary and enough for 95% discrete accuracy. • Continuous accuracy: it’s suggested to obtaining considerable continuous accuracy gains by increasing the panel size up to 6.02 Mb (95% continuous accuracy) or 2126 genes (cost of cost-effectiveness).
2. Somatic mutation detection	
(1) Analytical sensitivity	• Without other analytical needs, the 5% VAF cut-off for non-hotspot mutations is recommended for tissue sample assay to keep reliable somatic mutation results.
(2) Overall performance	• The recall and precision should be maintained at least above 0.8, or the difference between the reciprocal gap of recall and precision should be less than 0.179, which corresponds to the recall of 0.7 and precision of 0.8.
3. psTMB calculation rules	
(1) Inclusion or exclusion of mutations	• The synonymous, nonsense mutations and the hotspot filtering significantly affect the TMB accuracy. It’s recommended to involve the synonymous and nonsense mutation for psTMB calculation, without filtering hotspot mutations.
(2) VAF cut-off	• For tissue samples with at least 20% tumor purity, the 5% VAF cut-off is sufficient for psTMB calculation.
4. Classification method	
(1) Reference standard	• The indications of immunotherapy drugs approved by FDA and guidelines are the primary references.
(2) Classification stage	• The TMB results should be classified using estimated wesTMB values instead of psTMB values.
5. Metrics	
(1) Continuous accuracy	• The root mean squared logarithmic error (RMSLE) or alike metrics which could balance the influence of extreme values are suitable for the skewed TMB distribution.

## Methods

### Sample design

The four original cell line gDNA samples (A1, B1, C1, D1) used in this study originated from the 293T cell line and were edited to generate and accumulate random gene mutations^43^. The gDNA from the original 293T without induced mutations was used to simulated the paired normal sample. E1, the replicate of paired normal sample, was used as negative control tumor sample to measure the quality of germline sequencing and the reproducibility of panel assays. The gDNA from The gDNA materials of A1 and B1 were mixed with that of 293T in different proportions to simulate various tumor purities (Fig. 2a). A2, A3, A4, and A5 were derived from A1 with tumor purities of 20, 17.5, 15, and 12.5%. Similarly, B2. B3, B4, B5 and B6 originated from B1 with tumor purities of 40%, 20%, 17.5%, 15% and 12.5%, respectively. A3_2 and B6_2 served as the replicates of A3_1 and B6_1, respectively. The samples distributed to laboratories were human gDNA dissolved in colorless and transparent buffer, dispensed as 30 ul aliquots into 200 ul thin-wall polypropylene PCR tubes with a concentration of 30 ng/ul, and stored at −20 °C.

### Participating laboratories

Fifty-four laboratories participated in this multicenter study, submitting a total of 50 panel-based TMB results, which incorporated 38 unique panel methods (Fig. 2a). The methodological details were all collected, including targeted regions, wet-bench equipment and reagents, bioinformatic pipelines of somatic mutation detection, psTMB calculation rules and TMB classification methods, as well as their somatic mutation and TMB results on cell line gDNA samples.

### Whole exome sequencing and truth set

The truth set of somatic mutations and the reference values of wesTMB (rTMB) were established through four WES assays (Fig. 2b). Genetron (Beijing) carried out two tumor-normal paired assays on Illumina NovaSeq 6000 with PE300 mode, and MGI-Tech (Tianjin) carried out the other two tumor-normal paired assays on MGI DNBSEQ-T7 with PE100 mode. All assays were performed according to the manufacturer’s protocol.

#### Genetron WES assays

KAPA HiFi HotStart ReadyMix (2×), IDT ADAPTERS WITH ILLUMINA UDI CODES and Bechman Agencourt AMPure XP Kit were used for the cDNA library construction. SureSelect All Exon V5 kit and self-developed hybrid capture kit were used to capture the targeted cDNA segments. Agilent TapeStation DNA ScreenTape-D1000 and Qubit 4.0 were used for quality control. Then 200 ng of the cDNA library was submitted to sequencing on Illumina NovaSeq 6000 (mode = paired-end, reads length = 300, average depth = 600x, total targeted region size = 50 Mb). Trimmomatic v0.36 was used to refine the raw sequencing data (SLIDINGWINDOW = 4:15, LEADING = 3, TRAILING = 3, MINLEN = 36). Bwa v0.7.10-r789 and samtools v1.3 were used to align the raw data to hg19 genome. Picard v2.2.1 and GATK v3.5 with default settings were used to move the duplicated reads and recalibrate the base quality score. GATK Mutect v3.1-0-g72492bb and Strelka v2.9.2 were used to call the small somatic variants with tumor and normal samples. Then all variants were annotated using VEP v92 and filtered using self-developed scripts.

#### MGI-Tech WES assays

KAPA HIFI HOTSTART READYMIX and xGen® Hybridization and Wash Kit were used for the cDNA library construction, then the 96rxn xGen Exome Research Panel v1.0 was used for hybrid capture. BIOPTIC Qsep100 standard cartridge and Qubit 4.0 were used for quality control. 200 ng of the cDNA library was submitted to sequencing on MGI DNBSEQ-T7 (mode = paired-end, reads length = 100, average depth = 1000, total targeted region size = 51 Mb). UMI was applied to assure the performance of somatic mutation detection at low VAF level. After sequencing, SOAPnuke v2.0 was used for basic quality control and self-developed script was used to handle with the UMI results.9 Bwa mem v0.7.17-r1188 and samtools v 0.1.19-44428 cd were used to align the raw data to hg19 genome. Picard 2.18.1-1-g0d439ec-SNAPSHOT and GATK 4.0.8.1 with default settings were used to move the duplicated reads and recalibrate the base quality score. Then the SOMATK kit (developed by MGI) was used to call, annotate and filter the small somatic variants with tumor and normal samples.

#### Determination of credibility

We measured the quality and credibility of Genetron batch 1 (G1) and batch 2 (G2) and MGI-Tech batch 1 (M1) and batch 2 (M2) from following aspects: (1) sequencing quality (Q30, Q20, average sequencing depth, etc.), (2) reproducibility on repeated samples of same batch, (3) reproducibility on repeated samples of two batches, (4) the relationship of somatic mutation content and VAF among samples with different simulated tumor purity, (5) false positives on negative control sample. From these, we made sure that the somatic variants with at least 3 of the 4 WES assays could be considered as true positive, and variants with VAF not less than 9% is trustworthy.

#### Establishment of the truth set

Based on the credibility information, we used the somatic mutation results of the 4 WES assays to generate the truth set for 4 undiluted samples (A1, B1, C1, D1). Then average VAF value for each somatic mutation in truth set was calculated. By comparing the truth set and results of diluted samples, the truth set of the remaining samples were established. The reference wesTMB value was calculated as the average of wesTMB values of G1, M1 and M2, as the TMB result of G2 was not good.

#### TMB calculation

The parameters for wesTMB calculation followed the recommendation from the phase I of the FOCR TMB Harmonization Project^20^. Specifically, Missense_Mutation, In_Frame_Del, Nonsense_Mutation, In_Frame_Ins, Frame_Shift_Del and Frame_Shift_Ins variants with VAF ≥ 5%, t_depth > 25 and t_count > 3 were all included. Then samples were classified as TMB-L or TMB-H with a binary wesTMB cutoff at 10 mut/Mb.

### EQA data analysis

The submitted somatic mutation and TMB results from laboratories were evaluated using the truth set from reliable WES assays. Recall, precision and f1 score were employed to assess the somatic mutation result. RMSLE was utilized to measure the continuous accuracy of TMB results since it could minimize the potential bias from extreme high and low TMB values, as shown in Eq. (1). We evaluated the discrete accuracy of TMB results treating TMB-M and TMB-L as non-TMB-H. Accuracy was based on successful differentiation of TMB-H from non-TMB-H, as Eq. (2):

1 $${{\rm{RMSLE}}}=\sqrt{\frac{1}{{{n}}}\mathop{\sum }\limits_{i=1}^{n}{\left(\mathrm{ln}\left({{{\rm{eTMB}}}}_{i}+1\right)-\mathrm{ln}\left({{{\rm{rTMB}}}}_{i}+1\right)\right)}^{2}}$$

where eTMB refers to the estimated wesTMB value that was calculated using psTMB and psTMB-wesTMB regression model of submitted panel, while rTMB represents the reference wesTMB value.

2 $${{\rm{discrete}}}\,{{\rm{accuracy}}}=\frac{{{\rm{TP}}}+{{\rm{TN}}}}{{{\rm{TP}}}+{{\rm{TN}}}+{{\rm{FP}}}+{{\rm{FN}}}}$$

where true positive (TP) refers to samples that were consistently classified as TMB-H by WES and panel assays. True negative (TN), false positive (FP) and false negative (FN) can be determined similarly.

The impact of interaction between laboratory and panel methods on estimated wesTMB results was measured using the ordinary least squares model, type III ANOVA and Eta squared (*η*^2^). Details and codes are now available on Github.

### Dataset

We utilized the MC3 dataset containing numerous somatic mutations across various tumor types, which has been extensively used for method development and standardization of TMB assessment^20,44^. Somatic mutations were sourced from the mc3.v.0.2.8.PUBLIC.maf file on the Genomic Data Commons website (https://gdc.cancer.gov/about-data/publications/mc3-2017). Low-quality variants and samples were filtered out according to FILTER, t_depth, and other columns. The remaining variants were annotated with tumor purity and population databases. The wesTMB values of remaining samples in the dataset were then calculated and classified (Fig. 2a). The whole dataset was split into training, validation and testing sets in an 8:1:1 ratio for the in silico experiments.

### In silico experiment

The bed files containing the targeted regions of 38 unique panel methods served as the basic input. We constructed a technical parameter grid based on the real-world methodologies, including the recall and precision of somatic mutation detection, inclusion of mutations, filtering rules and the VAF cut-off for psTMB calculation. The filtering rules were also defined according to submitted methodologies.

A bed file and a set of parameters formed a synthetic panel-based TMB method. As the MC3 dataset was randomly split ten times in an 8:1:1 ratio, this simulated method was first applied to all the samples in the training dataset to calculate the psTMB results and generate ten simple psTMB-wesTMB linear models. Then it was applied to the validation dataset to select the optimal one, according to the *R*^2^, continuous and discrete accuracy. Finally, the performance of the complete synthetic panel method on the testing dataset was reported. The filtering rules expect hotspot filtering were all discarded since all the results with them were relatively bad. The details and codes of simulated test are available on Github.

### Assessment of feature importance

Three XGBoost-based regressor models were constructed after necessary feature engineering and collinearity check, using the results from simulated experiments. The XGBRegressors incorporated the following features: exonic size, gene count per Mb, rgbrp of somatic mutation detection, the inclusions of synonymous mutations, nonsense, nonstop, splicing site, and translation start site mutations, the filtering of hotspot mutations, along with the VAF cut-off for psTMB calculation. Based on computation rules of submitted panels, hotspot mutations were defined as mutations found in cancer hotspot or CIViC database, or with more than 20 records in COSMIC database. The other filtering rules were discarded after the pretest since the performance of results applying them were relatively worse compared to the those without them, which might be due to the lack of germline information in the MC3 dataset or inappropriate definition. The targeted values of the three XGBRegressors were the *R*^2^, RMSLE, and discrete accuracy, respectively. Root mean square error (RMSE) was utilized as the loss function. The SHapley Additive exPlanations (SHAP) approach was applied to explain the feature importance. The utilization of rgbrp to represent the accuracy of mutation detection will be further discussed in a later section titled “Individual factor evaluation and practice recommendations—Mutation detection accuracy”. Details and codes of feature engineering, collinearity check, hyperparameter optimization, model fitting and model explanation are all now available on the Github.

### Individual factor evaluation and practice recommendations

Certain discrete features exhibited interrelationships, such as the inclusion of mutations, while others like rgbrp and VAF cut-off were continuous with limited value ranges. As a result, XGBRegressors and SHAP values could only provide a general sense of feature importance without practical guidance. Hence, not only did we discuss the EQA results, but also conducted detailed analysis of the impacts of individual or combined features (technical parameters) on the TMB accuracy.

#### Panel size

In accordance with the common pattern of real panels, we compiled the genes covered by all participating panels and added them to the basic panel, starting from those covered by all panels and progressing to those tested by only one panel. Via this approach, we constructed over 40,000 simulated panels varying in exonic sizes and gene counts, which were then subjected to in silico experiments to demonstrate the relationship between panel size and the TMB accuracy. We used the MC3 dataset to identify the ideal methodological parameters and the corresponding performance for each unique panel (Fig. 2c). Since the targeted panels are not only designed to estimate TMB, but also to assist in cancer molecular typing and other targeted therapies by detecting driver mutations and actionable mutations, such as tyrosine kinase inhibitor and Poly (ADP-ribose) polymerase inhibitor, the targeted regions of participating panels were compared after filtering out the hotspot and driver gene, which were summarized from OncoKB, NCG and COSMIC database and listed in Supplementary Table 4. It should be mentioned that rather than creating more realistic panels by excluding specific exons, we included all the exons of the genes. That was because in real-world scenarios, there are other concerns when making tradeoffs regarding the targeted region.

#### Mutation detection accuracy

As a subset of the in silico experiment, the TMB accuracy of real panels with different assigned recall and precision values, from 0.5 to 1.0, were calculated.

In mainstream panel-based TMB methods, the somatic mutation detection accuracy inevitably influences the psTMB estimation, as TMB can be defined as Eq. (3):

3 $${{\rm{ps}}}{{\rm{TMB}}}=\frac{{{\rm{apparent}}}\,{{\rm{mutation}}}\,{{\rm{count}}}}{{{\rm{size}}}\,{{\rm{of}}}\,{{\rm{targeted}}}\,{{\rm{region}}}\,}$$

Considering recall and precision as the prevalent metrics for mutation detection and the number of true mutations in the genome region covered by the panel, the mutation count reported by the panel could be calculated as Eq. (4):

4 $$\begin{array}{ll}{{\rm{appear}}}\,{{\rm{mutation}}}\,{{\rm{count}}}={{\rm{true}}}\,{{\rm{mutations}}}+{{\rm{FP}}}-{{\rm{FN}}}\\ \qquad\qquad\qquad\qquad\qquad={{\rm{true}}}\,{{\rm{mutations}}}\times \left(1+\frac{1}{{{\rm{precision}}}}-\frac{1}{{{\rm{recall}}}}\right)\\ \qquad\qquad\qquad\qquad\qquad={{\rm{true}}}\,{{\rm{mutations}}}\times \left(1+{{\rm{rgbrp}}}\right)\end{array}$$

where FP refers to the reported mutation calls which are not in the truth set, and FN refers to the mutations that are present in the truth set but not reported by the laboratory.

This univariate representation of somatic mutation detection accuracy helps to eliminate the collinearity problem between recall and precision in the XGBRegressors, and clearly demonstrate the relationship between mutation detection accuracy and TMB accuracy. Specifically, the smaller the absolute value of rgbrp, the closer the number of mutations for psTMB calculation is to the number of true mutations, suggesting that perhaps the balance of detection strategy is crucial. Moreover, exchanging the same recall and precision values will yields different rgbrp values. Considering RMSLE punishing under-prediction more and the curse of dimensionality, cases only where recall ≥ precision were included.

#### Inclusion and filter of mutations

As a subset of the in silico experiment, the simulated parameter combinations with superior performance (e.g., higher *R*^2^, continuous and discrete accuracy than the other 95% parameter combinations) were extracted to robustly evaluate the importance of these rules. For each panel, the importance of one rule for one panel was determined by counting the times it appeared in the extracted parameter combinations. The overall importance of one rule was measured by the number of panels for which all the parameter combinations with top performance unanimously included or excluded it. The scripts with results are available on Github.

#### VAF cut-off

Samples in MC3 dataset with available tumor purity information were extracted for an additional in silico experiment to explore the TMB accuracy with different VAF cut-off values under conditions of 10–40% simulated tumor purity.

Ethical approval was waived because we used only publicly available data and commercial cell line materials in this study.

### Reporting summary

Further information on research design is available in the Nature Research Reporting Summary linked to this article.

### Supplementary information

Supplementary Information

Reporting Summary

## Data Availability

The raw FASTQ files of WES assays of gDNA samples in this study have been deposited in the Genome Sequence Archive for human database with the BioProject code PRJCA017746^45,46^. An online request is required for data download.

## Code availability

We used Python^47^ and R^48^ as the working languages; statsmodels^49^ for EQA data analysis; Polars^50^ for MC3 dataset processing; bedtools^51^ and pybedtools^52^ for in silico experiments; category_encoders^53^, scikit-learn^54^, XGBoost^55^ and SHAP^56^ for modeling and feature explanation; pandas^57^ and NumPy^58^ for data manipulation; matplotlib^59^, seaborn^60^, Jupyter Notebooks^61^, ggplot2^62^ and patchwork^63^ for data visualization. The custom scripts are all available along with the corresponding conda environments at https://github.com/YuanfengZhang/PANEL_TMB_STANDARDIZATION.

## References

[1] Vaddepally RK, Kharel P, Pandey R, Garje R, Chandra AB (2020). Review of indications of FDA-approved immune checkpoint inhibitors per NCCN guidelines with the level of evidence. Cancers.

[2] Kaushik I, Ramachandran S, Zabel C, Gaikwad S, Srivastava SK (2022). The evolutionary legacy of immune checkpoint inhibitors. Semin. Cancer Biol..

[3] Hellmann MD (2018). Tumor mutational burden and efficacy of nivolumab monotherapy and in combination with ipilimumab in small-cell lung cancer. Cancer Cell.

[4] Chan TA (2019). Development of tumor mutation burden as an immunotherapy biomarker: utility for the oncology clinic. Ann. Oncol..

[5] Cheng DT (2015). Memorial sloan kettering-integrated mutation profiling of actionable cancer targets (MSK-IMPACT): a hybridization capture-based next-generation sequencing clinical assay for solid tumor molecular oncology. J. Mol. Diagn..

[6] Milbury CA (2022). Clinical and analytical validation of FoundationOne®CDx, a comprehensive genomic profiling assay for solid tumors. PLoS ONE.

[7] Fenizia F (2021). Tumor mutation burden testing: a survey of the International Quality Network for Pathology (IQN Path). Virchows Arch..

[8] Sholl LM (2020). The promises and challenges of tumor mutation burden as an immunotherapy biomarker: a perspective from the International Association for the Study of Lung Cancer Pathology Committee. J. Thorac. Oncol..

[9] Fancello L, Gandini S, Pelicci PG, Mazzarella L (2019). Tumor mutational burden quantification from targeted gene panels: major advancements and challenges. J. Immunother. Cancer.

[10] Sung M-T, Wang Y-H, Li C-F (2022). Open the technical black box of tumor mutational burden (TMB): factors affecting harmonization and standardization of panel-based TMB. Int. J. Mol. Sci..

[11] Garofalo A (2016). The impact of tumor profiling approaches and genomic data strategies for cancer precision medicine. Genome Med..

[12] Buchhalter I (2019). Size matters: dissecting key parameters for panel-based tumor mutational burden analysis. Int. J. Cancer.

[13] Endris V (2019). Measurement of tumor mutational burden (TMB) in routine molecular diagnostics: in silico and real-life analysis of three larger gene panels. Int. J. Cancer.

[14] Stenzinger A (2019). Tumor mutational burden standardization initiatives: recommendations for consistent tumor mutational burden assessment in clinical samples to guide immunotherapy treatment decisions. Genes Chromosomes Cancer.

[15] Budczies J (2019). Optimizing panel-based tumor mutational burden (TMB) measurement. Ann. Oncol..

[16] Kazdal D (2019). Spatial and temporal heterogeneity of panel-based tumor mutational burden in pulmonary adenocarcinoma: separating biology from technical. Artifacts J. Thorac. Oncol..

[17] Quy PN (2019). Association between preanalytical factors and tumor mutational burden estimated by next‐generation sequencing‐based multiplex gene panel assay. Oncologist.

[18] Parikh K (2020). Tumor mutational burden from tumor-only sequencing compared with germline subtraction from paired tumor and normal specimens. JAMA Netw. Open.

[19] Bevins N, Sun S, Gaieb Z, Thorson JA, Murray SS (2020). Comparison of commonly used solid tumor targeted gene sequencing panels for estimating tumor mutation burden shows analytical and prognostic concordance within the cancer genome atlas cohort. J. Immunother. Cancer.

[20] Merino DM (2020). Establishing guidelines to harmonize tumor mutational burden (TMB): in silico assessment of variation in TMB quantification across diagnostic platforms: phase I of the Friends of Cancer Research TMB Harmonization Project. J. Immunother. Cancer.

[21] Budczies J (2020). Quantifying potential confounders of panel-based tumor mutational burden (TMB) measurement. Lung Cancer.

[22] Stenzinger A (2020). Harmonization and standardization of panel-based tumor mutational burden measurement: real-world results and recommendations of the quality in pathology study. J. Thorac. Oncol..

[23] Heydt C (2020). Analysis of tumor mutational burden: correlation of five large gene panels with whole exome sequencing. Sci. Rep..

[24] Heeke S (2020). Comparison of three sequencing panels used for the assessment of tumor mutational burden in NSCLC reveals low comparability. J. Thorac. Oncol..

[25] Zhang C, Wang H (2021). The source of the tumor tissue should be taken into consideration when distinguishing tumor mutational burden scores. Lung Cancer.

[26] Vega DM (2021). Aligning tumor mutational burden (TMB) quantification across diagnostic platforms: phase II of the Friends of Cancer Research TMB Harmonization Project. Ann. Oncol..

[27] Pang, J. et al. Benchmarking bioinformatics approaches for tumour mutational burden evaluation from a large cancer panel against whole-exome sequencing. *J. Clin. Pathol*. 10.1136/jcp-2022-208385 (2022). 10.1136/jcp-2022-208385 35906043

[28] Ramarao-Milne, P. et al. Comparison of actionable events detected in cancer genomes by whole-genome sequencing, in silico whole-exome and mutation panels. *ESMO Open* **7**, 100540 (2022). 10.1016/j.esmoop.2022.100540 PMC9463385 35849877

[29] Sun D (2022). Systematic assessment and optimizing algorithm of tumor mutational burden calculation and their implications in clinical decision-making. Front. Oncol..

[30] Esposito Abate R (2023). External quality assessment (EQA) for tumor mutational burden: results of an international IQN path feasibility pilot scheme. Virchows Arch..

[31] Valero C (2021). Response rates to anti-PD-1 immunotherapy in microsatellite-stable solid tumors with 10 or more mutations per megabase. JAMA Oncol..

[32] Zheng M (2022). Tumor mutation burden for predicting immune checkpoint blockade response: the more, the better. J. Immunother. Cancer.

[33] Sha D (2020). Tumor mutational burden as a predictive biomarker in solid tumors. Cancer Discov..

[34] Mankor JM (2020). Impact of panel design and cut-off on tumour mutational burden assessment in metastatic solid tumour samples. Br. J. Cancer.

[35] Li R (2020). Choosing tumor mutational burden wisely for immunotherapy: a hard road to explore. Biochim. Biophys. Acta Rev. Cancer.

[36] Liu N (2020). Progression of malignant pleural effusion during the early stage of gefitinib treatment in advanced EGFR-mutant lung adenocarcinoma involving complex driver gene mutations. Signal Transduct. Target. Ther..

[37] Xu Q (2022). Efficacy and safety of sintilimab plus anlotinib for PD-L1-positive recurrent or metastatic cervical cancer: a multicenter, single-arm, prospective phase II trial. J. Clin. Oncol..

[38] Lu C (2020). Association of genetic and immuno-characteristics with clinical outcomes in patients with RET-rearranged non-small cell lung cancer: a retrospective multicenter study. J. Hematol. Oncol..

[39] Wang D, Zhang Y, Li R, Li J, Zhang R (2023). Consistency and reproducibility of large panel next-generation sequencing: multi-laboratory assessment of somatic mutation detection on reference materials with mismatch repair and proofreading deficiency. J. Adv. Res..

[40] Xu H, DiCarlo J, Satya RV, Peng Q, Wang Y (2014). Comparison of somatic mutation calling methods in amplicon and whole exome sequence data. BMC Genomics.

[41] Mansfield AS, Peikert T, Vasmatzis G (2020). Chromosomal rearrangements and their neoantigenic potential in mesothelioma. Transl. Lung Cancer Res..

[42] Shi Y, Jing B, Xi R (2023). Comprehensive analysis of neoantigens derived from structural variation across whole genomes from 2528 tumors. Genome Biol..

[43] Peng R, Lin G, Li L, Li J (2022). Development of a novel reference material for tumor mutational burden measurement based on CRISPR/Cas9 technology. Front. Oncol..

[44] Ellrott K (2018). Scalable open science approach for mutation calling of tumor exomes using multiple genomic pipelines. Cell Syst..

[45] Chen T (2021). The genome sequence archive family: toward explosive data growth and diverse data types. Genomics Proteom. Bioinforma..

[46] CNCB-NGDC Members and Partners. (2023). Database resources of the National Genomics Data Center, China National Center for Bioinformation in 2023. Nucleic Acids Res..

[47] Van Rossum, G. & Drake Jr, F. L. *Python* (Centrum voor Wiskunde en Informatica Amsterdam, 1995).

[48] R Core Team. *R: A Language and Environment for Statistical Computing*. https://www.R-project.org/ (2021).

[49] Seabold, S. & Perktold, J. statsmodels: Econometric and statistical modeling with python. in *9th Python in Science Conference* (Scipy 2010, 2010).

[50] Vink, R. et al. pola-rs/polars: Python Polars 0.19.14. (Zenodo, 2023) 10.5281/zenodo.10150696.

[51] Quinlan AR, Hall IM (2010). BEDTools: a flexible suite of utilities for comparing genomic features. Bioinformatics.

[52] Dale RK, Pedersen BS, Quinlan AR (2011). Pybedtools: a flexible Python library for manipulating genomic datasets and annotations. Bioinformatcs.

[53] McGinnis WD, Siu C, S A, Huang H (2018). Category encoders: a scikit-learn-contrib package of transformers for encoding categorical data. J. Open Source Softw..

[54] Pedregosa F (2011). Scikit-learn: machine learning in Python. J. Mach. Learn. Res..

[55] Chen, T. & Guestrin, C. XGBoost: a scalable tree boosting system. in *Proceedings of the 22nd ACM SIGKDD International Conference on Knowledge Discovery and Data Mining* 785–794 10.1145/2939672.2939785 (2016).

[56] Lundberg, S. M. & Lee, S.-I. A unified approach to interpreting model predictions. in *Advances in Neural Information Processing Systems 30* (eds Guyon, I. et al.) 4765–4774 (Curran Associates, Inc., 2017).

[57] The pandas development team. pandas-dev/pandas: Pandas. v1.5.3 (Zenodo, 2023) 10.5281/zenodo.10107975.

[58] Harris CR (2020). Array programming with NumPy. Nature.

[59] Hunter JD (2007). Matplotlib: a 2D graphics environment. Comput. Sci. Eng..

[60] Waskom, M. L. seaborn: statistical data visualization. *J. Open Source Softw.* **6**, 3021 (2021).

[61] Kluyver, T. et al. *Jupyter Notebooks*—*A Publishing Format for Reproducible Computational Workflows* (eds Loizides, F. & Scmidt, B.) 87–90 (IOS Press, 2016).

[62] Wickham, H. *ggplot2: Elegant Graphics for Data Analysis* (Springer, 2016).

[63] Pedersen, T. L. *patchwork: The Composer of Plots.* https://patchwork.data-imaginist.com (2023).

