# Supplementary material for: Enhancing the quality of panel-based tumor mutation burden assessment: a comprehensive study of real-world and in-silico outcomes
Source: NPJ Precis Oncol. 2024 Jan 23;8:18. doi: 10.1038/s41698-024-00504-1 (PMC10805867; doi:10.1038/s41698-024-00504-1)
Supplement: Supplementary file 1 — Supplementary Information [file 41698_2024_504_MOESM1_ESM.pdf]

Supplementary Tables

Supplementary Table 1. Performance of 50 participating panel assays on gDNA samples

panel ID	Accuracy of somatic mutation detection				Accuracy of TMB estimation	Estimated wesTMB results					
	Recall	Precision	F1 score	FP on E1 (NC)	RMSLE	A1	B1	C1	D1	replicate of D1	E1
p01	0.994455	0.981752	0.988062	0	0.129195	97.9489	175.9106	120.2233	6.0654	6.0654	0.0000
p02	0.967742	0.949367	0.958466	0	0.141046	120.6000	164.2700	138.0700	5.6000	5.6000	0.0000
p03	0.989116	0.875376	0.928777	0	0.355431	158.2200	251.4200	142.4800	6.9100	6.9100	0.1000
p04	0.987557	0.843400	0.909804	0	0.328041	119.6600	229.4100	172.9100	7.3000	7.3000	0.0000
p05	0.738304	0.940410	0.827191	0	0.132513	87.0000	127.6000	111.5800	3.6800	3.6800	0.0000
p06	0.954635	0.910916	0.932263	0	0.155488	125.2637	165.2131	124.3444	6.2784	6.2690	0.0000
p07a	0.903621	0.857937	0.880187	0	0.313221	151.9786	216.0876	153.7594	6.8429	6.8429	0.0000
p07b	0.961702	0.887580	0.923155	0	0.313221	151.9786	216.0876	153.7594	6.8429	6.8429	0.0000
p07c	0.969681	0.869337	0.916771	0	0.301537	150.2000	214.3100	148.4200	6.8400	6.8400	0.0000
p07d	0.881320	0.865656	0.873418	0	0.309271	151.0882	217.8685	150.1978	6.8429	6.8429	0.0000
p08	0.682150	0.866711	0.763435	0	0.492892	122.1852	292.4193	248.4855	6.8657	6.1726	0.0000
p09	0.813525	0.921114	0.863983	0	0.406121	87.0900	141.3600	143.9500	3.9600	3.9600	1.3300
p10a	0.946988	0.921814	0.934231	0	0.192759	132.7502	179.8394	146.6669	5.4304	5.4304	0.0000
p10b	0.867925	0.908935	0.887956	0	0.202062	137.0339	184.1230	147.7430	4.3543	5.4304	0.0000
p10c	0.899351	0.917978	0.908569	1	0.227823	137.0339	175.5557	147.7430	6.4961	6.4961	0.0000
p10d	0.909237	0.918831	0.914009	0	0.231170	135.9600	179.8300	148.8000	6.4900	6.4900	0.0000
p10e	0.923567	0.915549	0.919540	0	0.208437	138.1099	180.9051	148.8087	5.4304	5.4304	0.0000
p11	0.900483	0.932933	0.916421	0	0.160588	120.9342	178.3140	127.6395	6.1646	6.1646	0.0000
p12	0.885385	0.928609	0.906482	0	0.080306	100.0000	132.2500	109.3000	5.0900	5.0900	0.1200
p13	0.878574	0.852090	0.865130	0	0.168755	105.8239	163.6284	113.0495	6.1111	6.1111	0.3307
p14	0.854651	0.919468	0.885876	0	0.058285	100.7414	142.5956	101.7622	4.2727	4.2727	0.0000
p15	0.609286	0.905010	0.728272	0	0.159410	121.1583	195.3995	132.0940	4.7100	5.4832	0.0000
p16	0.938062	0.891315	0.914091	0	0.122313	98.3600	152.8600	104.2200	6.3500	6.3500	0.0000
p17	0.930371	0.914602	0.922419	0	0.343094	141.3743	270.8078	161.6175	4.5798	4.5798	0.0000
p18	0.958416	0.971888	0.965105	0	0.279161	113.8000	232.7800	157.5600	6.3400	4.8000	0.0000
p19	0.679262	0.871477	0.763457	0	0.206117	84.7700	118.6200	83.8400	3.6200	3.6200	0.0000
p20	0.818369	0.901543	0.857945	0	0.213214	81.2028	118.7715	85.0072	6.0653	6.5408	0.0000
p21	0.882301	0.934396	0.907601	1	0.227605	111.9373	137.0046	113.6085	8.3258	7.7687	0.0000
p22	0.971607	0.949898	0.960630	0	0.354293	145.4197	243.2682	159.2213	7.3455	7.3455	-2.2615
p23a	0.877877	0.901157	0.889365	0	0.246636	110.3900	163.7100	117.8300	8.7100	8.7100	0.0000
p23b	0.883364	0.903088	0.893117	0	0.338240	135.8050	212.0650	150.0650	8.7100	8.7100	0.0000
p23c	0.884274	0.901951	0.893025	0	0.316726	110.3900	212.0650	150.0650	8.7100	8.7100	0.0000
p24	0.945109	0.909995	0.927219	0	0.184665	110.2500	173.4300	121.4600	7.2100	7.2100	0.0000
p25	0.895629	0.946277	0.920257	0	0.048732	106.7394	159.3503	117.2636	4.3456	4.3456	0.0000
p26	0.888188	0.943487	0.915002	0	0.132345	117.9170	182.0690	131.0530	5.0750	5.0750	0.0000
p27	0.715201	0.939832	0.812272	0	0.084010	106.9000	173.3240	124.0410	4.7560	4.7560	0.0000
p28	0.903465	0.915552	0.909468	0	0.181005	106.9469	150.9305	99.6098	7.3465	7.3465	0.0000
p29	0.989659	0.957000	0.973055	0	0.339196	132.5961	209.3547	142.1166	7.6403	8.2354	0.5000
p30	0.849481	0.864437	0.856894	0	0.330063	91.0265	73.5661	92.6138	5.3122	5.3122	0.0000
p31	0.933613	0.903987	0.918561	0	0.228987	85.1000	128.1700	85.1000	7.1500	7.1500	-0.2700
p32a	0.939198	0.923664	0.931366	0	0.064476	94.8400	144.4400	104.8400	5.2400	5.2400	-0.3600
p32b	0.937418	0.921290	0.929284	0	0.087223	103.5600	158.1100	114.9000	5.8200	5.8200	-0.2900
p33	0.889791	0.915274	0.902353	0	0.427794	154.5600	302.1900	181.2800	5.6500	6.2900	0.0000
p34	0.755713	0.841351	0.796236	0	0.519630	163.7800	327.9100	230.3600	5.0700	5.0700	0.0000
p35a	0.921891	0.874882	0.897771	3	0.100830	107.4023	178.3881	125.7366	4.9902	5.5089	0.0000
p35b	0.829787	0.929678	0.876897	0	0.076099	98.0335	155.0243	106.2655	5.5816	5.5816	0.0000
p36	0.750000	0.787037	0.768072	0	0.161093	98.9398	160.8244	106.9032	7.0244	7.0244	0.0000
p37a	0.993964	0.856088	0.919888	0	0.642255	166.4028	394.0130	288.8144	5.1623	5.1623	0.0000
p37b	0.996190	0.848108	0.916204	0	0.641240	166.8965	397.9494	284.3843	5.1623	5.1623	0.0000
p38	0.821670	0.900990	0.859504	0	0.125902	101.3700	150.4700	100.2900	3.3900	3.3900	0.0000

Supplementary Table 2. Methodology details of submitted panel-based TMB assays

	Panel Design				Wet bench						Dry bench			
Panel ID	Exonic size (Mb)	Gene count	Method name	Source	DNA shearing	UMI	Sequencing mode	Reads length (bp)	Sequencing depth (x)	Sequencing platform	Reference genome	Somatic mutation callers	VAF cut-off of somatic mutation	VAF cut-off of TMB calculation
p01	0.6	500	NA	self-developed	Ultrasonic_Covaris	No	Paired-End	100	500	Gene+Seq 2000	hg19	GATK Mutect2, Manta, VarScan2	5.0%	5.0%
p02	0.7	518	NA	self-developed	Ultrasonic_Covaris	No	Paired-End	100	300	MGISEQ-2000	hg19	GATK Mutect2	1.0%	5.0%
p03	0.8	1460	He'ji'an	Genelotus	Ultrasonic_Covaris	Applied	Paired-End	150	1500	Illumina NovaSeq 6000	hg19	Lofreq, Samtools, self-developed	1.0%	2.5%
p04	0.9	437	Geneseeq Prime	Geneseeq	Ultrasonic_Bioruptor	Applied	Paired-End	150	1500	Illumina NovaSeq 6000	hs37d5	Delly, self-developed	1.0%	2.0%
p05	1.0	639	ALLNGS Panel 639	YUNYING MEDICINE	Ultrasonic_Covaris	No	Paired-End	150	1000	Illumina NextSeq 500	GRCh37	GATK Mutect2, VariDict, self-developed	1.0%	5.0%
p06	1.0	769	baishibo	Genecast	Ultrasonic_Covaris	No	Paired-End	200	500	Illumina NovaSeq 6000	hg19	GATK Mutect2, Samtools, VarDict, VarScan2, self-developed, other	2.0%	5.0%
p07a	1.0	1021	Onco1021plus	Gene+	Ultrasonic_Covaris	Applied	Paired-End	100	500	MGI DNBSEQ-T7RS	hs37d5	GATK HaplotypeCaller, GATK Mutect2, Samtools, self-developed	5.0%	5.0%
p07b	1.0	1021	Onco1021plus	Gene+	Ultrasonic_Bioruptor	Applied	Paired-End	100	500	Gene+Seq 2000	hs37d5	GATK HaplotypeCaller, GATK Mutect2, Samtools, self-developed	5.0%	5.0%
p07c	1.0	1021	Onco1021plus	Gene+	Ultrasonic_Bioruptor	Applied	Paired-End	100	500	Gene+Seq 2000	hs37d5	GATK HaplotypeCaller, GATK Mutect2, Samtools, self-developed	5.0%	5.0%
p07d	1.0	1021	Onco1021plus	Gene+	Ultrasonic_Covaris	Applied	Paired-End	100	500	Gene+Seq 2000	hs37d5	GATK HaplotypeCaller, GATK Mutect2, Samtools, self-developed	5.0%	5.0%
p08	1.1	889	NA	self-developed	Enzyme_TIANGEN	No	Paired-End	300	1000	Illumina NovaSeq 6000	GRCh37	GATK Mutect2, self-developed	1.0%	1.0%
p09	1.1	500	NA	self-developed	Ultrasonic_Covaris	No	Paired-End	150	1000	Illumina NextSeq 500	hg19	VarScan2	5.0%	5.0%
p10a	1.1	518	OncoScreen Plus	Burning Rock	Ultrasonic_Covaris	No	Paired-End	150	1000	Illumina NextSeq 500	b37	VarDict, self-developed	5.0%	5.0%
p10b	1.1	520	OncoScreen Plus	Burning Rock	Ultrasonic_Covaris	No	Paired-End	150	1000	Illumina NextSeq 500	b37	VarDict, self-developed	5.0%	5.0%
p10c	1.1	520	OncoScreen Plus	Burning Rock	Ultrasonic_Covaris	No	Paired-End	150	1000	Illumina NextSeq 550Dx	b37	VarDict, Provided by Manufacturer	5.0%	5.0%
p10d	1.1	520	OncoScreen Plus	Burning Rock	Ultrasonic_Covaris	No	Paired-End	150	1000	Illumina NextSeq 550Dx	b37	VarDict, self-developed	5.0%	5.0%
p10e	1.1	520	OncoScreen Plus	Burning Rock	Ultrasonic_Covaris	No	Paired-End	150	1000	Illumina NextSeq 550	b37	VarDict, self-developed	5.0%	5.0%
p11	1.3	600	predicineATLAS	preidicine	Ultrasonic_Bioruptor	Similar tech applied	Paired-End	150	2000	Illumina NovaSeq 6000	humanG1Kv37	GATK Mutect2, self-developed	5.0%	5.0%
p12	1.4	680	NA	self-developed	Enzyme_self-developed	No	Paired-End	300	500	Illumina NovaSeq 6000	hg19	GATK HaplotypeCaller, VarScan2, self-developed	1.0%	5.0%
p13	1.4	808	XianagYi plus	AcornMed	Ultrasonic_Covaris	No	Paired-End	150	1000	Illumina NovaSeq 6000	hg19	GATK HaplotypeCaller, GATK Mutect2	1.0%	5.0%
p14	1.4	559	NA	self-developed	Ultrasonic_Covaris	Applied	Paired-End	300	2000	Illumina NovaSeq 6000	hg19	self-developed	5.0%	5.0%
p15	1.4	551	OncoPrint 551	BIOTECAN	Ultrasonic_Covaris	No	Paired-End	75	500	Illumina NextSeq CN500	hg19	GATK Mutect2, Samtools, VariDict, self-developed	2.0%	5.0%
p16	1.4	583	NA	self-developed	Ultrasonic_Qsonica	Applied	Paired-End	300	1000	Illumina NovaSeq 6000	hs37d5	GATK HaplotypeCaller, GATK Mutect2, Manta, self-developed	5.0%	5.0%
p17	1.4	599	NA	self-developed	Ultrasonic_Bioruptor	No	Paired-End	150	1000	Illumina NextSeq 2000	hg19	GATK Mutect2, VariDict, VarScan2	5.0%	3.0%
p18	1.5	800	NA	self-developed	Enzyme_Other	No	Paired-End	150	1000	Illumina NovaSeq 6000	hg19	VarDict, self-developed, other	1.0%	2.5%
p19	1.5	645	NA	self-developed	Ultrasonic_Bioruptor	No	Paired-End	150	800	Illumina NovaSeq 6000	hg19	GATK HaplotypeCaller, GATK Mutect2, Samtools, self-developed, other	1.0%	5.0%
p20	1.6	551	NA	self-developed	Enzyme_KAPA	Similar tech applied	Paired-End	150	1000	Illumina NovaSeq 6000	hg19	VarDict	2.0%	5.0%
p21	1.6	580	NA	self-developed	Enzyme_self-developed	No	Paired-End	150	1000	Illumina NextSeq 500	hg19	VarDict	5.0%	5.0%
p22	1.6	667	RRA plus	Repugene	Ultrasonic_Branson	No	Paired-End	150	500	Illumina HiSeq X10	hg19	Delly, GATK Mutect2, Manta	1.0%	2.5%
p23a	1.6	601	NA	Daan	Enzyme_QIAGEN	No	Paired-End	150	5000	Illumina NovaSeq 6000	hg19	Delly, Samtools, self-developed	2.0%	5.0%
p23b	1.6	601	NA	Daan	Enzyme_QIAGEN	No	Paired-End	150	5000	Illumina NovaSeq 6000	hg19	Samtools, VarScan2	2.0%	5.0%
p23c	1.6	601	NA	Daan	Enzyme_QIAGEN	No	Paired-End	150	5000	Illumina NovaSeq 6000	hg19	Samtools, VarScan2	2.0%	5.0%
p24	1.6	654	NA	BerryOncology	Enzyme_self-developed	Applied	Paired-End	150	1000	Illumina NovaSeq 6000	hg19	self-developed	3.0%	5.0%
p25	1.7	641	NA	self-developed	Ultrasonic_Covaris	No	Paired-End	150	1000	Illumina NovaSeq 6000	hg19	Delly, GATK Mutect2, VarScan2	2.0%	2.0%
p26	1.7	641	NA	self-developed	Enzyme_KAPA	No	Paired-End	150	800	Illumina NovaSeq 6000	hg19	Delly, GATK Mutect2, VarScan2	5.0%	5.0%
p27	1.7	641	NA	self-developed	Enzyme_KAPA	No	Paired-End	150	800	Illumina NovaSeq 6000	hg19	Delly, GATK Mutect2, VarScan2	2.0%	2.0%
p28	1.8	1267	YuceOne Plus	YUCEBIO	Ultrasonic_LongLight	No	Paired-End	100	500	MGI DNBSEQ-T7	hg19	Delly, GATK HaplotypeCaller, Samtools, VarDict	2.0%	5.0%
p29	1.8	1576	NA	self-developed	Ultrasonic_Covaris	No	Paired-End	150	1000	Illumina NovaSeq 6000	hg19	Freebayes, GATK Mutect2, VarDict	5.0%	5.0%
p30	1.9	576	NA	self-developed	Ultrasonic_Covaris	No	Paired-End	150	1000	Illumina Nextseq 2000	hg19	Delly, GATK HaplotypeCaller, Samtools, TNscope, self-developed	5.0%	5.0%
p31	2.0	623	NA	self-developed	Ultrasonic_Other	No	Paired-End	150	300	Illumina NovaSeq 6000	hs37d5	GATK Mutect2, Manta, other	5.0%	5.0%
p32a	2.1	830	OncoPanScan	Genetron	Ultrasonic_Covaris	No	Paired-End	300	1000	Illumina NovaSeq 6000	b37	GATK Mutect2, Samtools, self-developed	5.0%	10.0%
p32b	2.1	830	OncoPanScan	Genetron	Ultrasonic_Covaris	No	Paired-End	200	1000	Genetron S2000	b37	Samtools, self-developed	5.0%	5.0%
p33	2.1	641	NA	self-developed	Ultrasonic_Covaris	Applied	Paired-End	100	500	MGISEQ-2000	hg19	GATK Mutect2, VarScan2	5.0%	3.0%
p34	2.3	683	NA	self-developed	Ultrasonic_Qsonica	Applied	Paired-End	300	1000	Illumina NovaSeq 6000	hg19	Freebayes, GATK HaplotypeCaller, GATK Mutect2, self-developed	1.0%	2.0%
p35a	2.6	578	NA	USCI	Enzyme_KAPA	No	Paired-End	150	1000	Illumina NextSeq 550	hg19	VarDict	5.0%	5.0%
p35b	2.6	592	NA	USCI	Ultrasonic_Covaris	No	Paired-End	100	2000	MGISEQ-2000RS	hg19	Samtools, VarScan2	1.0%	5.0%
p36	2.6	565	NA	self-developed	Enzyme_Nanodigmbio	No	Paired-End	100	2000	MGISEQ-2000	hg19	Lofreq	5.0%	5.0%
p37a	2.9	688	Oseq	MGI	Ultrasonic_Covaris	Applied	Paired-End	100	900	MGISEQ-2000	hg19	GATK HaplotypeCaller, Samtools, self-developed	1.0%	1.5%
p37b	2.9	688	Oseq	MGI	Ultrasonic_Covaris	Applied	Paired-End	100	900	MGISEQ-2000	hg19	GATK HaplotypeCaller, Samtools, self-developed	1.0%	1.5%
p38	4.5	890	NA	self-developed	Ultrasonic_Covaris	Applied	Paired-End	300	1000	MGI DNBSEQ-T7	hg19	Delly, Samtools, other	5.0%	5.0%

Supplementary Table 2 (continued)

Panel ID	Dry bench							Reference standard(s) for eastablishing the cutoff	Associated Researches	
	Involved variant classifications	Applied filtering rules	Germline strategy	Classification stage	Classification method	TMB-H Cut-off1	TMB-H Cut-off2		Associated Clinical Researches	Performance Researches
p01	Nonsense, Missense, Nonstop, Splicing Site, Indel	none	tumor-normal	unstated	Binary	10.00		wesTMB		
p02	Nonsense, Missense, Nonstop, Indel	Other	tumor-normal	eTMB (wesTMB)	Binary	10.00		wesTMB		
p03	Nonsense, Missense, Nonstop, Splicing Site, Indel	SNP	tumor-normal	psTMB	Binary	10.93		wesTMB		
p04	Synonymous, Nonsense, Missense, Nonstop, Splicing Site, Indel	SNP, Driver	tumor-normal	psTMB	Binary	5.48		Clinical outcomes, wesTMB	PMC7542666,PMC7874268,PMC7160902,PMC10638181,PMC10334452,PMC9773983,PMC8236808,PMC8248503,PMC7773813,PMC9054482,PMC10483597	https://www.cmde.org.cn//xwdt/shpbg/20231027130554151.html
p05	Synonymous, Nonsense, Missense, Splicing Site, Indel	SNP, Driver	tumor-normal	eTMB (wesTMB)	Binary	10.00		Clinical outcomes, wesTMB	PMC10582542,PMID: 36346122	
p06	Nonsense, Missense, Nonstop, Splicing Site, Indel, Other	SNP, Driver	tumor-normal	psTMB	Binary	7.20		Other	PMC10587944,PMC10401588,PMC10301745,PMC9005141,PMC10121773,PMC10373867,PMID: 37261522	
p07a	Synonymous, Nonsense, Missense, Nonstop, Splicing Site, Indel	SNP, Driver, Known, Other	tumor-normal	psTMB	Binary	9.52		Other	PMC7657842,PMC9652899,PMC6755545,PMC7959707, PMC6448263,PMC8663088,PMC6448263,PMC6607931	
p07b	Synonymous, Nonsense, Missense, Nonstop, Splicing Site, Indel	SNP, Driver, Known, Other	tumor-normal	psTMB	Binary	9.52		Other	as above	
p07c	Synonymous, Nonsense, Missense, Nonstop, Splicing Site, Indel	SNP, Driver, Known, Other	tumor-normal	psTMB	Binary	9.52		Other	as above	
p07d	Synonymous, Nonsense, Missense, Nonstop, Splicing Site, Indel	SNP, Driver, Known, Other	tumor-normal	psTMB	Binary	9.52		Other	as above	
p08	Nonsense, Missense, Nonstop, Splicing Site, Indel	none	tumor-normal	unstated	Binary	8.24		wesTMB		
p09	Synonymous, Nonsense, Missense, Indel	SNP, Driver	tumor-normal	eTMB (wesTMB)	Binary	10.00		Clinical outcomes		
p10a	Nonsense, Missense, Nonstop, Splicing Site, Indel, Other	SNP	tumor-normal	eTMB (wesTMB)	Binary	10.00		Other	PMC8096980,PMC9335993,PMC7900143,PMC7082297, PMC7604404,PMC8350084,PMC9052533,PMC9184574, PMC10394813,PMC7250885,PMC9148684,PMID: 34933103,PMID: 36184068	PMID: 35305253
p10b	Nonsense, Missense, Nonstop, Splicing Site, Indel, Other	SNP	tumor-normal	unstated	Binary	10.00		Other	as above	as above
p10c	Nonsense, Missense, Nonstop, Splicing Site, Indel, Other	SNP	tumor-normal	unstated	Binary	10.00		Other	as above	as above
p10d	Nonsense, Missense, Nonstop, Splicing Site, Indel, Other	SNP	tumor-normal	psTMB	Binary	6.14		Other	as above	as above
p10e	Nonsense, Missense, Nonstop, Splicing Site, Indel, Other	SNP	tumor-normal	unstated	Binary	10.00		Other	as above	as above
p11	Nonsense, Missense, Nonstop, Splicing Site, Indel	Driver	tumor-normal	eTMB (wesTMB)	Binary	10.00		wesTMB	PMC9418247,PMC10107199,PMC10150240,PMID: 37584165,PMID: 37584165	
p12	Synonymous, Nonsense, Missense, Nonstop, Splicing Site, Indel	none	tumor-normal	eTMB (wesTMB)	Binary	10.00		Clinical outcomes	PMC8056263	
p13	Synonymous, Nonsense, Missense, Nonstop, Splicing Site, Indel	SNP, Other	tumor-normal	psTMB	Binary	10.16		Other	34729830	
p14	Synonymous, Nonsense, Missense, Nonstop, Splicing Site, Indel	SNP, Hotspot	tumor-normal	psTMB	Binary	5.80		wesTMB		
p15	Nonsense, Missense, Nonstop, Splicing Site, Indel	Other	tumor-normal	psTMB	Binary	8.63		wesTMB		
p16	Nonsense, Missense, Nonstop, Splicing Site, Indel	SNP, Driver, Hotspot	tumor-normal	eTMB (wesTMB)	Binary	10.00		wesTMB		
p17	Synonymous, Nonsense, Missense, Nonstop, Splicing Site, Indel	SNP, Known	tumor-normal	eTMB (wesTMB)	Binary	10.00		Clinical outcomes		
p18	Nonsense, Missense, Nonstop, Indel	SNP, Driver	tumor-normal	psTMB	Binary	10.33		Other		
p19	Nonsense, Missense, Nonstop, Splicing Site, Indel, Other	SNP, Hotspot	tumor-normal	psTMB	Binary	14.54		Other		
p20	Nonsense, Missense, Nonstop, Indel	SNP, Driver, Hotspot, Other	tumor-normal	psTMB	Binary	8.82		Other		
p21	Nonsense, Missense, Splicing Site, Indel	SNP, Driver, Hotspot	tumor-normal	eTMB (wesTMB)	Binary	10.00		FDA panels		
p22	Synonymous, Nonsense, Missense, Nonstop, Splicing Site, Indel	SNP, Hotspot	tumor-normal	psTMB	Binary	7.40		wesTMB		
p23a	Nonsense, Missense, Nonstop, Splicing Site, Indel	SNP	tumor-normal	eTMB (wesTMB)	Binary	10.40		wesTMB		
p23b	Nonsense, Missense, Nonstop, Splicing Site, Indel	SNP	tumor-normal	eTMB (wesTMB)	Binary	10.00		wesTMB		
p23c	Nonsense, Missense, Nonstop, Splicing Site, Indel	SNP	tumor-normal	psTMB	Binary	9.34		wesTMB		
p24	Synonymous, Nonsense, Missense, Nonstop, Splicing Site, Indel	SNP, Hotspot	tumor-normal	psTMB	Binary	7.75		Other	PMID: 35433492, PMID: 37141396, PMID: 37587479	
p25	Nonsense, Missense, Nonstop, Indel	SNP, Driver, Known	tumor-normal	psTMB	Binary	9.04		wesTMB		
p26	Nonsense, Missense, Nonstop, Indel	SNP, Driver, Known	tumor-normal	psTMB	Binary	10.08		wesTMB		
p27	Nonsense, Missense, Nonstop, Indel	SNP, Driver, Known	tumor-normal	psTMB	Binary	10.37		wesTMB		
p28	Nonsense, Missense, Nonstop, Splicing Site, Indel	none	tumor-normal	psTMB	Binary	8.45		Clinical outcomes, wesTMB	PMID: 37086484, PMID: 36425562, PMID: 35613927	
p29	Nonsense, Missense, Nonstop, Splicing Site, Indel	SNP, Driver, Other	tumor-normal	psTMB	Binary	11.05		Clinical outcomes, wesTMB, FDA panels		
p30	Nonsense, Missense, Nonstop, Splicing Site, Indel	Driver, Known	tumor-normal	psTMB	Binary	10.23		wesTMB		
p31	Nonsense, Missense, Nonstop, Indel	SNP, Hotspot, Known	tumor-normal	eTMB (wesTMB)	Ternary	10.00	20.00	wesTMB		
p32a	Nonsense, Missense, Nonstop, Splicing Site, Indel	SNP, Hotspot	tumor-normal	psTMB	Binary	8.04		FDA panels	PMID: 36999792, PMID: 35396765, PMID: 35480123, PMID: 36033514	PMID: 37477357
p32b	Nonsense, Missense, Nonstop, Splicing Site, Indel, Other	Hotspot	tumor-normal	psTMB	Binary	8.88		FDA panels	as above	as above
p33	Synonymous, Nonsense, Missense, Nonstop, Splicing Site, Indel	Driver	tumor-normal	psTMB	Binary	6.92		wesTMB		
p34	Nonsense, Missense, Nonstop, Splicing Site, Indel	SNP	tumor-normal	eTMB (wesTMB)	Binary	14.52		wesTMB		
p35a	Synonymous, Nonsense, Missense, Nonstop, Splicing Site, Indel	SNP, Driver, Hotspot, Other	tumor-normal	psTMB	Binary	6.68		Other		
p35b	Nonsense, Missense, Nonstop, Splicing Site, Indel	SNP	tumor-normal	psTMB	Binary	7.54		FDA panels		
p36	Nonsense, Missense, Nonstop, Indel	SNP, Driver	tumor-normal	psTMB	Binary	8.19		Other		
p37a	Synonymous, Nonsense, Missense, Nonstop, Splicing Site, Indel, Other	SNP, Driver, Hotspot	tumor-normal	psTMB	Ternary	6.15	10.57	Other	PMID: 33777741, PMID: 35865459, PMID: 37743841, PMID: 36291846, PMID: 36942084, PMID: 37089387	
p37b	Synonymous, Nonsense, Missense, Nonstop, Splicing Site, Indel, Other	SNP, Driver, Hotspot	tumor-normal	psTMB	Ternary	6.15	10.57	Other	as above	
p38	Nonsense, Missense, Nonstop, Splicing Site, Indel	SNP	tumor-normal	eTMB (wesTMB)	Binary	10.00		Clinical outcomes, FDA panels		

Supplementary Table 3. Results of statistic tests in this article

Panel groups to compare	Equality of variances		Performance difference			
	Levene statistic	p-value	Method	t/u-value	p-value	Effect size (abs)
including nonstop mutations vs. not	0.085	0.772	Mann-Whitney U test	62.000	0.744	0.103
including splice site mutations vs. not	0.880	0.353	Mann-Whitney U test	231.000	0.245	0.510
inculding synonymous mutations vs. not	2.403	0.128	Student's t-test (standard)(two-sided)	2.199	0.033	0.611
filtering SNPs muations vs. not	0.018	0.894	Mann-Whitney U test	233.000	0.431	0.200
filtering driver muations vs. not	1.660	0.204	Student's t-test (standard)(two-sided)	1.237	0.222	0.347
filtering hotspot muations vs. not	1.111	0.297	Student's t-test (standard)(two-sided)	-0.034	0.973	0.008
filtering known muations vs. not	0.512	0.478	Mann-Whitney U test	212.000	0.780	0.013
utilized in clinical studies vs. not	0.251	0.618	Student's t-test (standard)(two-sided)	0.254	0.801	0.013

Supplementary Table 4. Full list of hotspot and driver genes

ACVR1	A1CF	ABI1	ABL1	ABL2	ACKR3	LCK	KNSTRN	KRAS	LARP4B	LATS1	LATS2
AKT1	ACVR1B	ACVR2A	AFDN	AFF3	AFF4	LRIG3	LEF1	LEPROTL1	LMO1	LMO2	LPP
APC	AKT2	AKT3	ALK	AMER1	ANK1	MAFB	LRP1B	LYL1	LZTR1	MACC1	MAF
ARHGEF10	APOBEC3B	AR	ARAF	ARHGAP26	ARHGAP5	MAP3K1	MALT1	MAML2	MAP2K1	MAP2K2	MAP2K4
ARNT	ARHGEF10L	ARHGEF12	ARID1A	ARID1B	ARID2	MECOM	MAP3K13	MAPK1	MAX	MDM2	MDM4
ATP2B3	ASXL1	ASXL2	ATF1	ATM	ATP1A1	MLF1	MED12	MEN1	MET	MGMT	MITF
BAP1	ATR	ATRX	AXIN1	AXIN2	B2M	MSH2	MLH1	MLLT10	MN1	MPL	MRTFA
BCL11B	BARD1	BAX	BAZ1A	BCL10	BCL11A	MYB	MSH6	MSI2	MTCP1	MTOR	MUTYH
BCL9L	BCL2	BCL2L12	BCL3	BCL6	BCL9	MYOD1	MYC	MYCL	MYCN	MYD88	MYH9
BLM	BCLAF1	BCOR	BCORL1	BIRC3	BIRC6	NCOA4	N4BP2	NAB2	NBEA	NBN	NCOA2
BRD3	BMP5	BMPR1A	BRAF	BRCA1	BRCA2	NFATC2	NCOR1	NCOR2	NDRG1	NF1	NF2
CACNA1D	BRD4	BRIP1	BTG1	BTK	BUB1B	NOTCH2	NFE2L2	NFKB2	NFKBIE	NKX2-1	NOTCH1
CASP8	CALR	CAMTA1	CARD11	CARS1	CASP3	NSD3	NPM1	NR4A3	NRAS	NRG1	NSD2
CBLC	CASP9	CBFA2T3	CBFB	CBL	CBLB	NUP98	NT5C2	NTHL1	NTRK1	NTRK2	NTRK3
CCND3	CCDC6	CCNB1IP1	CCNC	CCND1	CCND2	PATZ1	NUTM1	OLIG2	P2RY8	PABPC1	PALB2
CD28	CCNE1	CCR4	CCR7	CD209	CD274	PDCD1LG2	PAX3	PAX5	PBRM1	PBX1	PCBP1
CDH10	CD74	CD79A	CD79B	CDC73	CDH1	PHOX2B	PDGFB	PDGFRA	PDGFRB	PER1	PHF6
CDKN1A	CDH11	CDH17	CDK12	CDK4	CDK6	PLAG1	PIK3CA	PIK3CB	PIK3R1	PIK3R2	PIM1
CHD2	CDKN1B	CDKN2A	CDKN2C	CDX2	CEBPA	POLE	PLCG1	PML	PMS1	PMS2	POLD1
CLTC	CHD4	CHEK2	CHST11	CIC	CIITA	PPARG	POLG	POLQ	POT1	POU2AF1	POU5F1
CPEB3	CLTCL1	CNBD1	CNBP	CNOT3	COL2A1	PRDM2	PPM1D	PPP2R1A	PPP6C	PRDM1	PRDM16
CRNKL1	CREB1	CREB3L1	CREB3L2	CREBBP	CRLF2	PRPF40B	PREX2	PRF1	PRKACA	PRKAR1A	PRKCB
CTNNB1	CRTC1	CSF1R	CSF3R	CTCF	CTNNA2	PTPN13	PSIP1	PTCH1	PTEN	PTK6	PTPN11
CYLD	CTNND1	CTNND2	CUL3	CUX1	CXCR4	PTPRT	PTPN6	PTPRB	PTPRC	PTPRD	PTPRK
DDB2	CYP2C8	CYSLTR2	DAXX	DCAF12L2	DCC	RAF1	QKI	RAC1	RAD17	RAD21	RAD51B
DDX6	DDIT3	DDR2	DDX10	DDX3X	DDX5	RECQL4	RANBP2	RAP1GDS1	RARA	RB1	RBM10
DNMT3A	DEK	DGCR8	DICER1	DNM2	DNMT1	RHOA	REL	RET	RFWD3	RGPD3	RGS7
EIF1AX	DROSHA	EBF1	ECT2L	EED	EGFR	ROS1	RHOH	RIT1	RMI2	RNF43	ROBO2
EP300	EIF3E	ELF3	ELF4	ELK4	ELL	RUNX1	RPL10	RPL22	RPL5	RSPO2	RSPO3
ERBB3	EPAS1	EPHA3	EPHA7	EPS15	ERBB2	SDHAF2	RUNX1T1	S100A7	SALL4	SBDS	SDHA
ERG	ERBB4	ERCC2	ERCC3	ERCC4	ERCC5	SETD1B	SDHB	SDHC	SDHD	SET	SETBP1
ETV6	ESR1	ETNK1	ETV1	ETV4	ETV5	SGK1	SETD2	SETDB1	SF3B1	SFPQ	SFRP4
FAM47C	EWSR1	EXT1	EXT2	EZH2	FAM135B	SKI	SH2B3	SH3GL1	SIRPA	SIX1	SIX2
FANCG	FANCA	FANCC	FANCD2	FANCE	FANCF	SMARCB1	SLC34A2	SMAD2	SMAD3	SMAD4	SMARCA4
FBXO11	FAS	FAT1	FAT3	FAT4	FBLN2	SOCS1	SMARCD1	SMARCE1	SMC1A	SMO	SND1
FEV	FBXW7	FCGR2B	FCRL4	FEN1	FES	SRC	SOX2	SOX21	SOX9	SPEN	SPOP
FHIT	FGFR1	FGFR2	FGFR3	FGFR4	FH	STAG1	SRSF2	SRSF3	SSX1	SSX2	SSX4
FLT4	FKBP9	FLCN	FLI1	FLNA	FLT3	STK11	STAG2	STAT3	STAT5B	STAT6	STIL
FOXP1	FOXA1	FOXL2	FOXO1	FOXO3	FOXO4	TAL2	SUFU	SUZ12	SYK	TAF15	TAL1
GATA2	FOXR1	FSTL3	FUBP1	FUS	GATA1	TEC	TBL1XR1	TBX3	TCF3	TCF7L2	TCL1A
GPC3	GATA3	GLI1	GNA11	GNAQ	GNAS	TFEB	TENT5C	TERT	TET1	TET2	TFE3
H3C2	GPC5	GRIN2A	GRM3	H3-3A	H3-3B	TNFAIP3	TGFBR2	TLX1	TLX3	TMEM127	TNC
HMGA2	HEY1	HIF1A	HIP1	HLF	HMGA1	TRAF7	TNFRSF14	TNFRSF17	TP53	TP63	TPM3
HOXC11	HNF1A	HNRNPA2B1	HOXA11	HOXA13	HOXA9	TSC2	TRIM24	TRIM27	TRIM33	TRRAP	TSC1
IDH1	HOXC13	HOXD11	HOXD13	HRAS	ID3	USP8	TSHR	U2AF1	UBR5	USP44	USP6
IL7R	IDH2	IGF2BP2	IKBKB	IKZF1	IL6ST	WRN	VAV1	VHL	WAS	WIF1	WNK2
JAK2	IRF4	IRS4	ISX	ITGAV	JAK1	YWHAE	WT1	WWTR1	XPA	XPC	XPO1
KCNJ5	JAK3	JUN	KAT6A	KAT6B	KAT7	ZNF429	ZBTB16	ZEB1	ZFHX3	ZMYM3	ZNF331
KIT	KDM5A	KDM5C	KDM6A	KDR	KEAP1	ZNF479	ZNF521	ZNRF3	ZRSR2		
KNL1	KLF4	KLF6	KMT2A	KMT2C	KMT2D						

Supplementary Figures & Legends

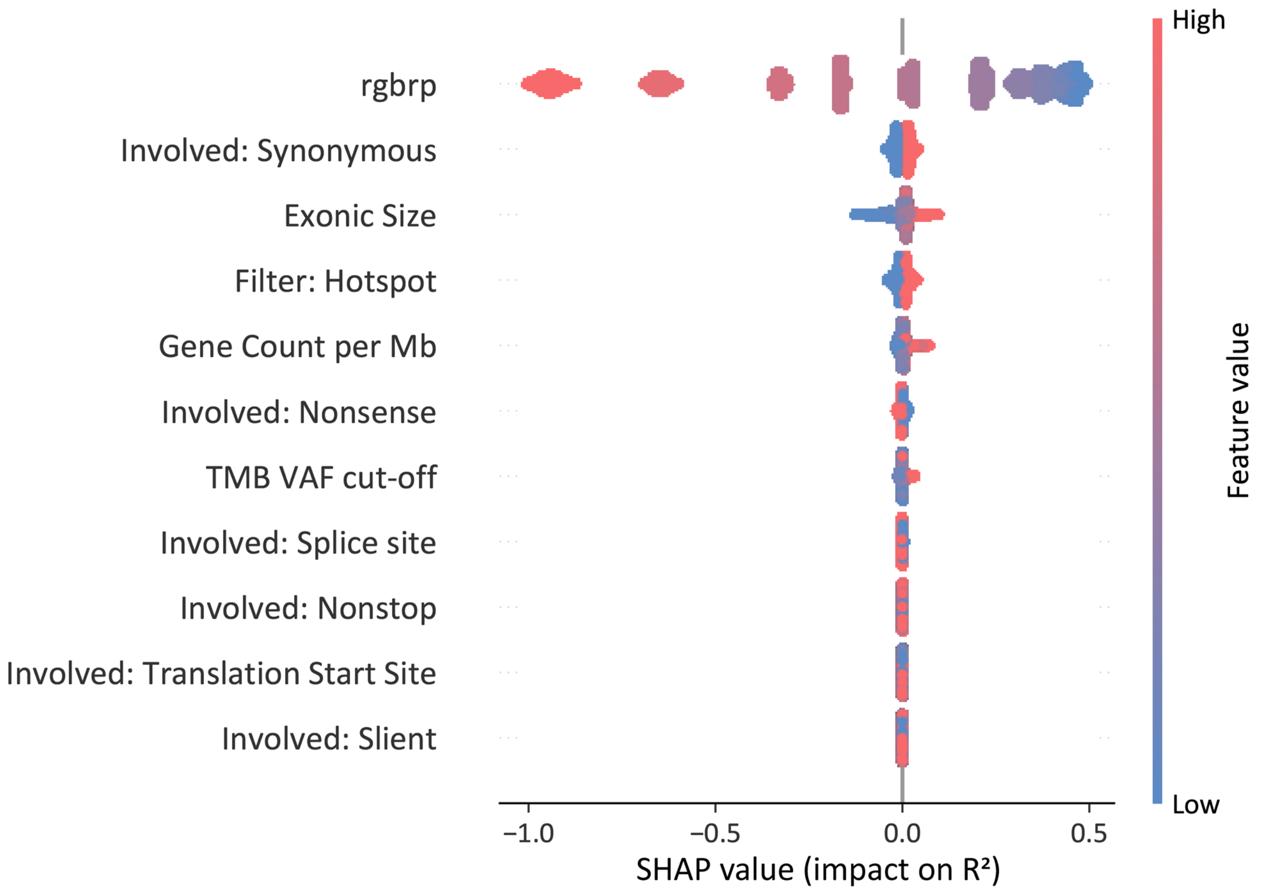

Supplementary Fig 1. SHAP values show the relative importance of these technical parameters as features in the XGBRegressor model according to the R^2.
